# Donor after circulatory death in pancreas transplantation: a scoping review of the literature

**DOI:** 10.3389/frtra.2025.1517354

**Published:** 2025-02-25

**Authors:** Mathieu Chassot, Marc Scheen, Charles-Henri Wassmer, Philippe Compagnon, Andrea Peloso, Fadi Haidar

**Affiliations:** ^1^ Faculté de médecine, Université de Genève, Genève, Switzerland; ^2^ Service de néphrologie et transplantation, Hôpitaux Universitaires de Genève, Genève, Switzerland; ^3^ Service de chirurgie viscérale et transplantation, Hôpitaux Universitaires de Genève, University of Geneva, Genève, Switzerland

**Keywords:** pancreas transplantation, donor after circulatory death, pancreas shortage, postoperative outcomes, normothermic regional perfusion, pancreatic graft thrombosis, delayed graft function, warm ischemia time

## Abstract

The growing disparity between the demand for pancreas transplants and the availability of suitable organs underscores the urgent need for innovative donor strategies, including the utilization of donors after circulatory death (DCD). This scoping review presents a comprehensive comparative analysis of transplantation outcomes between DCD and donors after brain death (DBD), focusing on pancreatic graft survival, postoperative complications, and functional metrics such as graft performance and HbA1c levels. Although DCD grafts were suspected to be associated with higher rates of early complications, including delayed graft function and thrombosis, altogether resulting from potentially more ischemia-reperfusion injuries, their long-term outcomes are comparable to those of DBD grafts. This equivalence is likely driven by careful donor selection, a meticulous pancreas procurement, use of normothermic regional perfusion and a short ischemic time. The findings highlight the transformative potential of DCD donors in expanding the pancreas donor pool, addressing critical organ shortages, and enhancing transplant accessibility. This review advocates for the integration of DCD donors into routine clinical practice, emphasizing the need for optimized clinical protocols and organ allocation strategies. By leveraging DCD donors more effectively, the transplant community can make significant strides in improving patient outcomes and addressing the global organ shortage crisis.

## Introduction

Simultaneous pancreas and kidney transplantation (SPK) remains the gold standard treatment for patients with type 1 diabetes (T1D) who have progressed to end-stage renal disease (1). This combined procedure not only restores normal pancreatic endocrine functions but also eliminates the need for exogenous insulin therapy while significantly enhancing recipients' quality of life (1). For select patients with T1D or insulin-requiring type 2 diabetes (T2D) without underlying advanced chronic kidney disease, pancreas transplant alone (PTA) serves as an alternative therapeutic strategy (2). Epidemiologically speaking, PTA is used less frequently and accounts for less than 10% of all pancreatic transplants performed in the world (2). Since the pioneering pancreas transplants of the 1960s, advancements in surgical techniques, immunosuppressive protocols, and patient selection criteria have markedly improved transplantation success rates, with graft survival at one year of 90.7% for SPK (3, 4). Despite these achievements, the persistent shortage of suitable organ donors continues to pose a formidable challenge, particularly for PTA. Addressing this critical limitation has led to increased utilization of pancreases from donors after circulatory death (DCD). However, DCD transplantation is associated with inherent challenges, including extended warm ischemia times and reduced organ quality compared to traditional donors after brain death (DBD). Indeed, several groups reported similar results after pancreas transplantation from DCD and DBD donors (5–7). The negative impact of a DCD donor can be overcome by careful donor selection and the use of normothermic regional perfusion (NRP) (8–10). These improvements have mitigated the impact of warm ischemia and enhanced the metabolic function of grafts, leading to a gradual rise in the number of DCD pancreas transplants worldwide (10, 11). This trend reflects the growing expertise and confidence within the transplant community in addressing the unique challenges posed by DCD donation. Transplant centers worldwide share a growing interest for DCD donation and globally improving DCD transplant related outcomes and post-operative care. This scoping review aims to critically evaluate the efficacy and safety of pancreas transplants derived from DCD donors. By analyzing outcomes such as graft survival, post-operative complications, and long-term metabolic function, this scoping review aims to establish evidence-based guidelines and update improvements in current transplantation protocols. Finally, the findings aim to promote the effective integration of DCD organs into routine clinical practice, thereby expanding the donor pool and addressing the critical organ shortage in pancreas transplantation.

This review aims to investigate and compare pancreas transplantation outcomes between DCD and DBD donors using comparative analysis with a broad review of the literature and by means of a data-driven methodological approach. Furthermore, we aimed to clarify and gather the scarce and heterogenous data in the field of DCD pancreas transplantation which could have significant implications for clinical practice and patient management in transplantation medicine.

### DCD and DBD donors in pancreas transplantation

Pancreas procurement for transplantation involves two primary methods: Donation After Circulatory Death (DCD) and Donation After Brain Death (DBD), each with specific processes, implications for organ viability, and outcomes. DCD involves organ donation following the irreversible cessation of circulatory and respiratory functions, further categorized into controlled DCD, where life support withdrawal is planned, and uncontrolled DCD, where cardiac arrest occurs unexpectedly. In contrast, DBD occurs when a patient is declared dead based on neurological criteria, with the absence of brain activity while cardiovascular functions are maintained through artificial support. This distinction highlights the differing criteria for confirming death: cardio-respiratory cessation in DCD vs. neurological cessation in DBD (8).

The procurement process for DCD donors is inherently complex, requiring careful coordination to balance ethical, clinical, and logistical considerations. After the decision to withdraw life support, the potential DCD donor is monitored until circulatory arrest occurs. A mandatory standoff period of 2–5 min is commonly used in most centers to confirm death, adhering to ethical guidelines and following consultation with the donor's family. After this period of “no touch”, absence of cardiac activity by ultrasound is confirmed and absence of cerebral reflex is assessed in order to confirm the death of the donor. Only after this confirmation, does organ retrieval begin. This process introduces an unavoidable warm ischemic time (WIT), during which the pancreas remains at body temperature without perfusion until preservation measures are initiated (8) or NRP is started (12).

In contrast, the procurement process for DBD donors is more controlled. Brain death is declared while artificial respiratory and hemodynamic functions are maintained, allowing organ perfusion to continue until organs retrieval. This preservation of perfusion reduces ischemic injury and provides a more favorable environment for organ quality and function. The duration and nature of ischemia significantly influence the quality and viability of organs procured from DCD and DBD donors. In DBD, circulation is maintained allowing a precise dissection and preparation of the organs until aortic canulation and preservation solution perfusion. As soon as the aorta is canulated and clamped, immediate cold perfusion with preservation solutions can be apply and the organ can be cooled down without a warm ischemic phase. Cold ischemic times (CIT) range from 6 to 18 h. This controlled environment minimizes ischemic damage, leading to better preservation of the pancreas (8).

Conversely, DCD pancreases experience varying periods of WIT. After therapeutic withdrawal, the time between the arterial hypotension takes place, the death of the donor and until the cold perfusion starts is critical and should be as short as possible. The median functional WIT for DCD pancreases is approximately 29 min, and prolonged WIT increases the risk of ischemia-reperfusion injury, potentially resulting in delayed graft function, graft pancreatitis, thrombosis, or primary non-function (13, 14).

Differences in donor characteristics further impact organ quality and preservation. DBD donors are often younger individuals with fewer comorbidities, providing higher-quality organs and leading to superior immediate and long-term graft outcomes (13, 15). These donors are typically declared brain dead due to traumatic brain injuries or severe neurological conditions, ensuring organ viability is preserved under optimal conditions.

In contrast, DCD donors frequently present with more complex medical histories, such as cardiovascular disease or anoxia, which may compromise organ function and quality. Additionally, the withdrawal of life support introduces variability in ischemia duration, contributing to the challenges of using DCD organs for transplantation (8).

**Figure 1:**
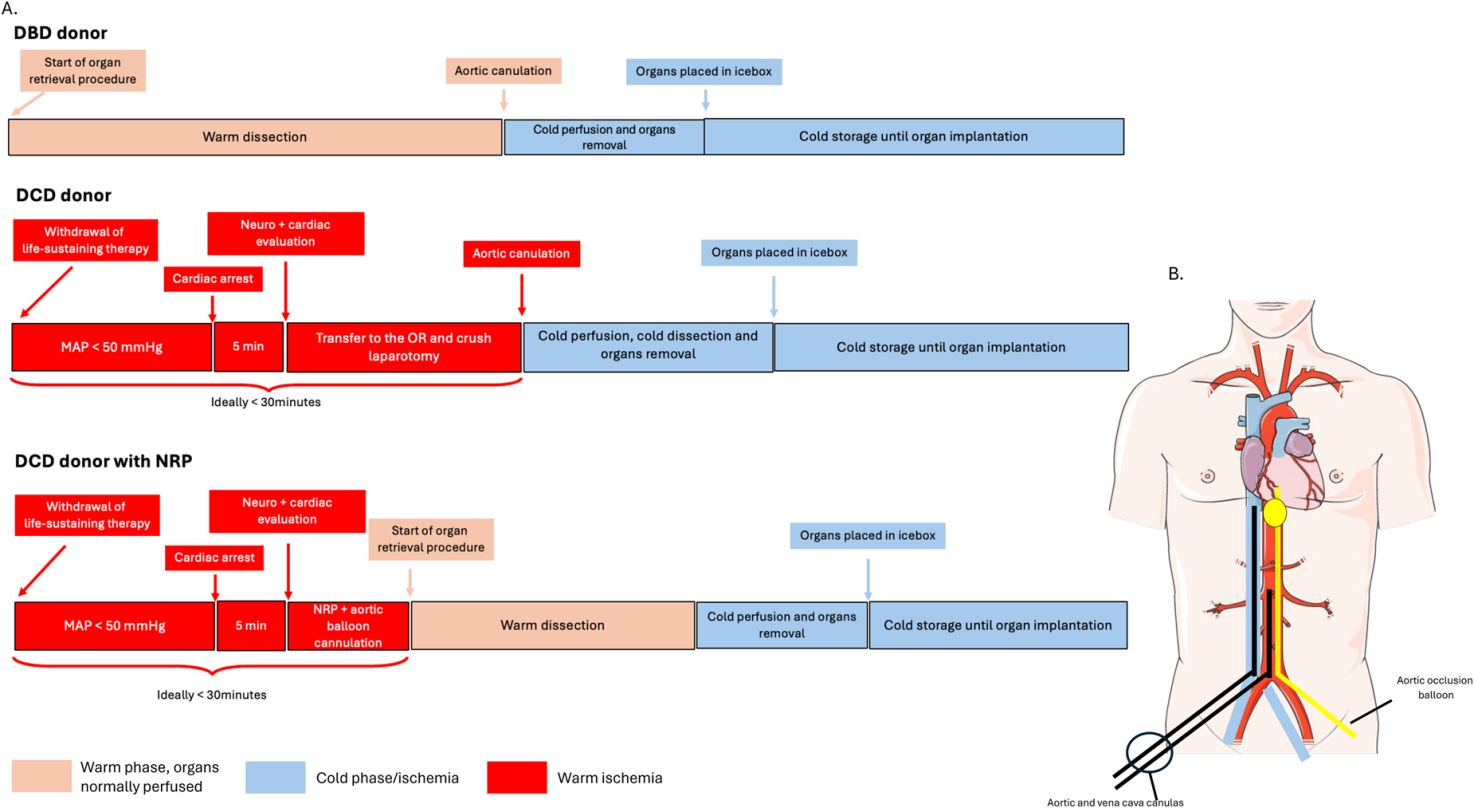
DBD and DCD procedures and steps. **(A)** DBD, DCD and DCD with NRP procedures. Neuro and cardiac evaluation are defined by neurological brain reflexes and cardiac ultrasound allowing the confirmation of brain death and absence of cardiac activity, respectively. **(B)** Schematic representation of the NRP cannulas (black) and occlusion balloon (yellow). MAP: mean arterial pressure, OR: operating room.

The outcomes of pancreas transplantation vary between DCD and DBD donors due to these differences in procurement processes and organ quality. DBD pancreas grafts are associated with higher utilization rates, superior graft function, and lower complication rates compared to DCD grafts. Some studies report pancreas transplant utilization rates from DCD donors as low as 4%, whereas DBD utilization rates are significantly higher (8). However, when donor characteristics are comparable between DCD and DBD donors, no differences are observed in term of post-transplantation graft survival (7). Furthermore, advancements in preservation techniques with perfusion machines have demonstrated significant improvements for marginal organs and organ retrieved from DCD donors, especially for the kidney and the liver. However, despite promising pre-clinical results for the pancreas, there is at the moment no perfusion machine used for the pancreas in clinical setting (Figure 1).

## Materials and methods

### Research strategy

In this scoping review, a comprehensive search strategy was employed to ensure the inclusion of the most relevant and high-quality studies. Several major electronic databases were systematically explored, including PubMed, the Cochrane Library, and Google Scholar.

The search was conducted using a combination of MeSH terms and free-text keywords to ensure a thorough capture of pertinent studies. The MeSH terms included “pancreas transplantation,” “cause of death,” “death, sudden, cardiac,” “brain death,” “host vs. graft reaction,” and “treatment outcome.” These controlled vocabulary terms were complemented by an array of free-text keywords to encompass studies not indexed under MeSH terms, including “cardiac death,” “brain death,” “circulatory death,” “heart-beating,” “non-heart-beating,” “outcome,” and “survival.”

### Inclusion and exclusion criteria

Studies were included if they addressed the outcomes of pancreas transplantation in donors after circulatory death (DCD) compared to those after brain death (DBD). Included publication ranged from original research articles, cohort studies, observational studies, to meta-analyses. Publications that did not compare outcomes between DCD and DBD donors, as well as case reports or studies with inadequate methodologies, were excluded. Specific attention was given to the documented warm ischemia time (WIT) in studies that described outcomes among DCD transplantation given its documented importance in determining pancreas allograft outcomes.

### Study selection process

**Figure 2:**
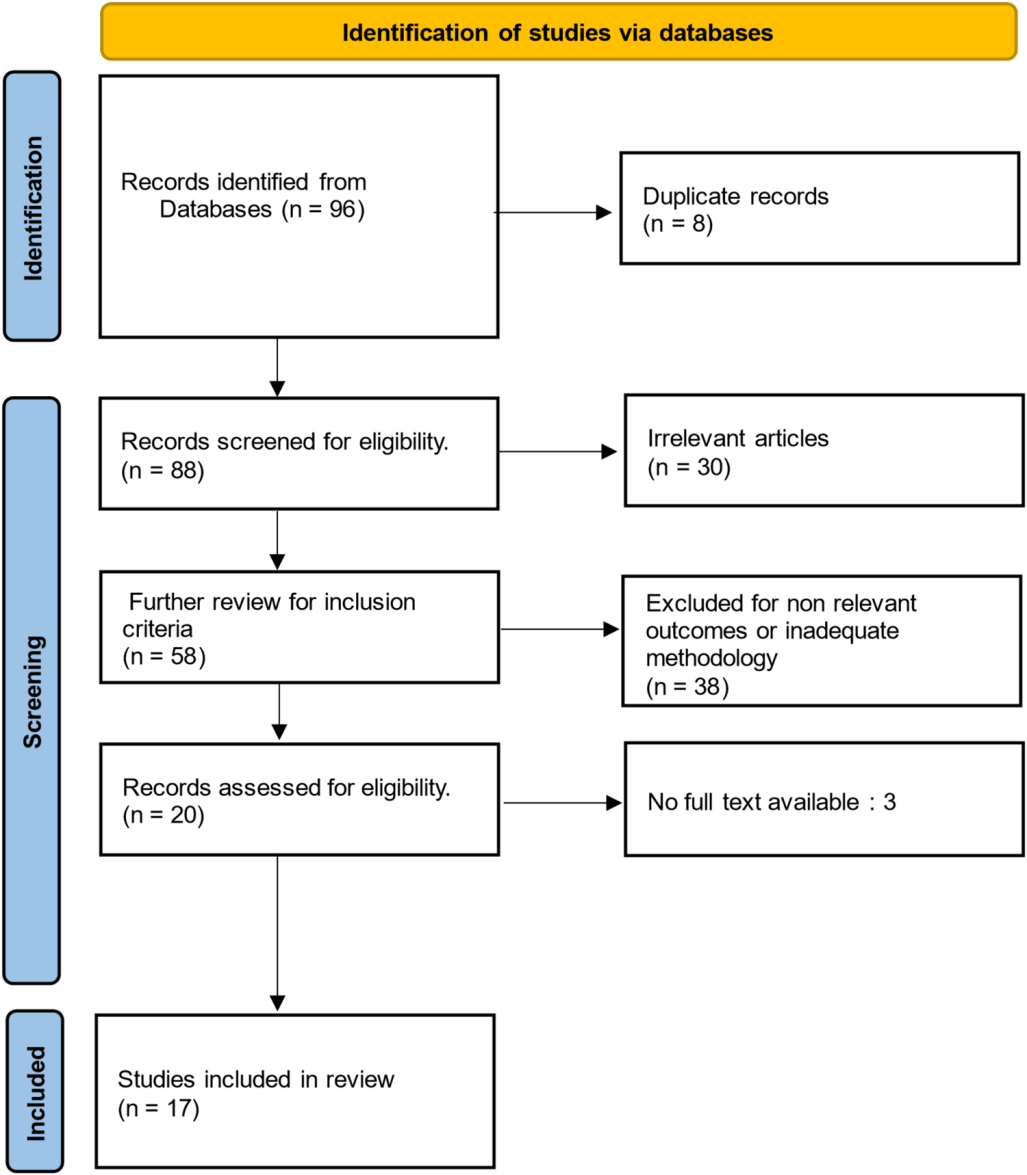
PRISMA diagram. This diagram delineates the sequential stages of study identification, screening through abstract review, assessment of full-text eligibility, and ultimate inclusion in the scoping review (Figure 2).

A PRISMA flow chart was utilized to systematically outline the study selection process (Figure 2).

### Data extraction and quality assessment

Relevant data regarding outcomes in pancreas transplantation were collected manually from selected studies. This collection process included details about study design, participant characteristics, and key results such as patient survival, graft survival, and reported complications. The analysis focused on examining significant trends and comparing outcomes between donors after circulatory death (DCD) and brain death (DBD). The primary goal was to identify and understand critical differences that could influence transplantation practices. This approach has enabled a thorough synthesis of the data, highlighting the practical and theoretical implications of the findings for pancreas transplantation.

### Data synthesis

Given the anticipated limited availability of data on DCD donors in PTA, coupled with the heterogeneity in study designs and reported outcomes, a scoping review was deemed the most appropriate format for this analysis. This approach allowed for the identification and synthesis of key trends and patterns across the available literature. Conducting a meta-analysis was considered unsuitable due to the significant variability in study methodologies and outcomes.

## Results

Data and outcomes concerning pancreas transplantation from Donation after Circulatory Death (DCD) and Donation after Brain Death (DBD) donors, were obtained from 17 different studies (6, 16–34). Key endpoints include graft survival, complication rates, and metabolic outcomes such as HbA1c levels, providing a comparative evaluation of transplant quality between DCD and DBD donors. Particular emphasis is placed on the impact of ischemia time -especially warm ischemia- on transplant success. Here, “Warm Ischemia Time” (WIT) is defined as the interval from the withdrawal of life-sustaining treatment (WLST) to the initiation of cold preservation perfusion.

### Pancreatic graft survival rate

In the largest cohort studies reviewed, five-year graft survival rates were consistently comparable between donation after circulatory death (DCD) and donation after brain death (DBD) groups. These findings were exemplified by Callaghan et al. (16), encompassing 403 DCD transplants, and Muthusamy et al. (17), which reported outcomes for 134 DCD transplants. Similar conclusions were drawn in more recent investigations. In 2023, Bleszynski et al. (18) analyzed 32 DCD transplants, and Richards et al. included 72 DCD transplants (19). Notably, no significant differences in graft survival were observed at 1, 3, 5, or 10 years post-transplantation. Additionally, Leemkuil et al. (20) suggested that these consistent outcomes can be achieved by addressing and mitigating other risk.

### Pancreatic graft thrombosis

**Figure 3:**
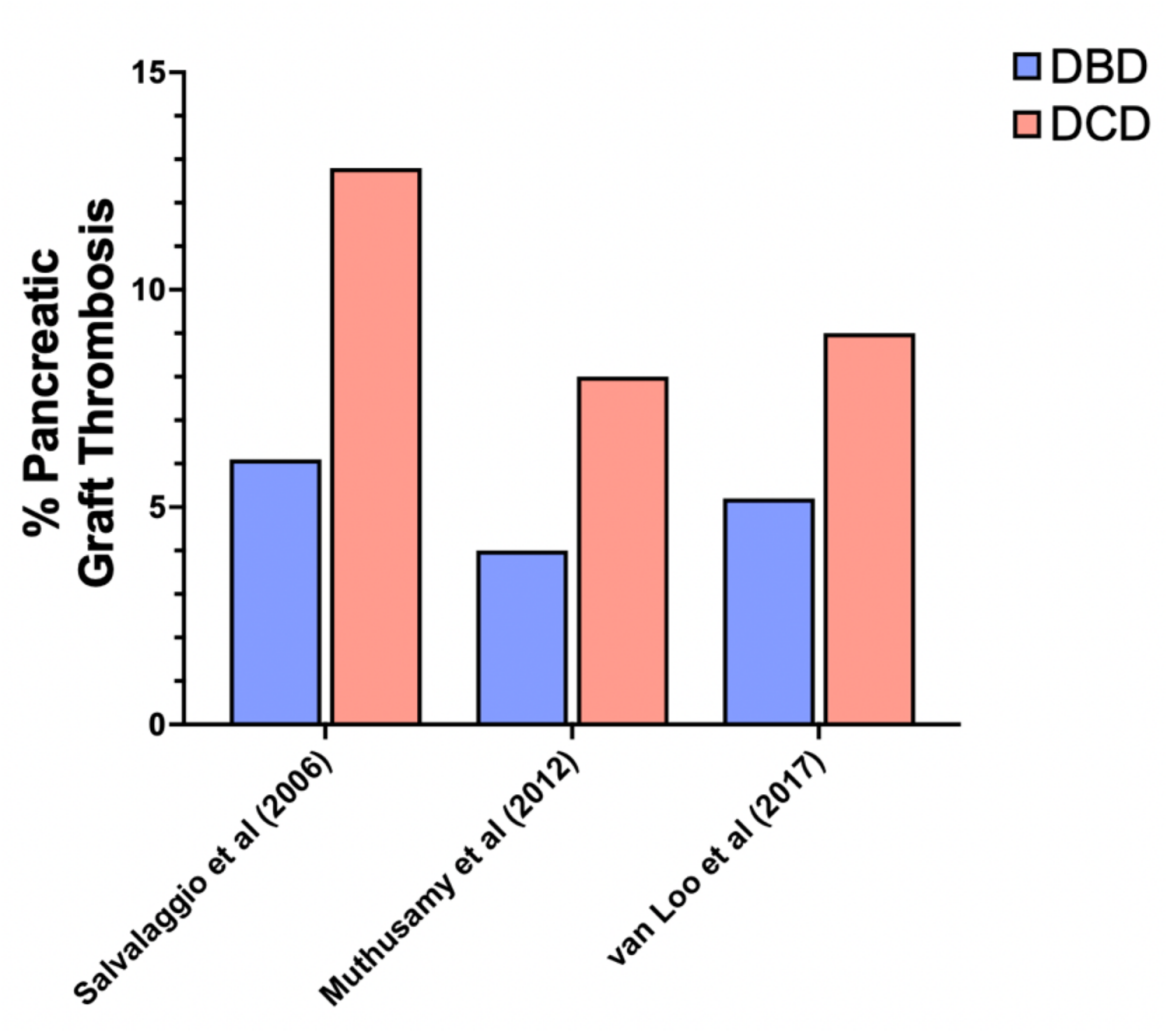
Pancreatic graft thrombosis. Incidence rates of pancreatic graft thrombosis reported in the literature for DCD and DBD pancreatic allografts.

Pancreatic graft thrombosis is a critical concern in pancreas transplantation, with varying reports regarding its incidence in DBD vs. DCD grafts (17, 21–23). Key references highlighted a potentially higher risk of graft thrombosis in DCD pancreas transplants. In 2017, a meta-analysis by Mittal et al. (21) suggested that DBD pancreas transplantation is associated with a lower rate of graft thrombosis compared to DCD pancreas transplantation, even if not translated into a significant difference in graft survival. Muthusamy et al. (17) and Salvalaggio et al. (22), raised concerns about worse outcomes in DCD pancreas transplants, with extrapolations drawn from DCD kidney transplantation data. In 2006, they used data from the OPTN/UNOS Registry, focusing on graft failure due to graft thrombosis. Among 47 DCD and 2,431 DBD simultaneous pancreas-kidney, vascular thrombosis was the leading cause of graft failure and occurred more frequently in DCD SPK transplants (12.8%) compared to DBD transplants (6.1%, *p* = 0.06). In 2012 Muthusamy et al. (17) compared all the pancreas transplantation performed in UK between 2006 and 2010. A total of 1,009 pancreas transplants were analyzed including 134 from DCD and 875 from DBD donors. Pancreatic graft thrombosis, was more frequent in DCD grafts (8% vs. 4% for DBD grafts). Van Loo et al. published a systematic review with a meta-analysis, and reported a higher thrombosis rate in DCD grafts (9%) compared to DBD grafts (5.2%), particularly in elderly recipients (23) (Figure 3).

### Delayed graft function (DGF)

**Figure 4:**
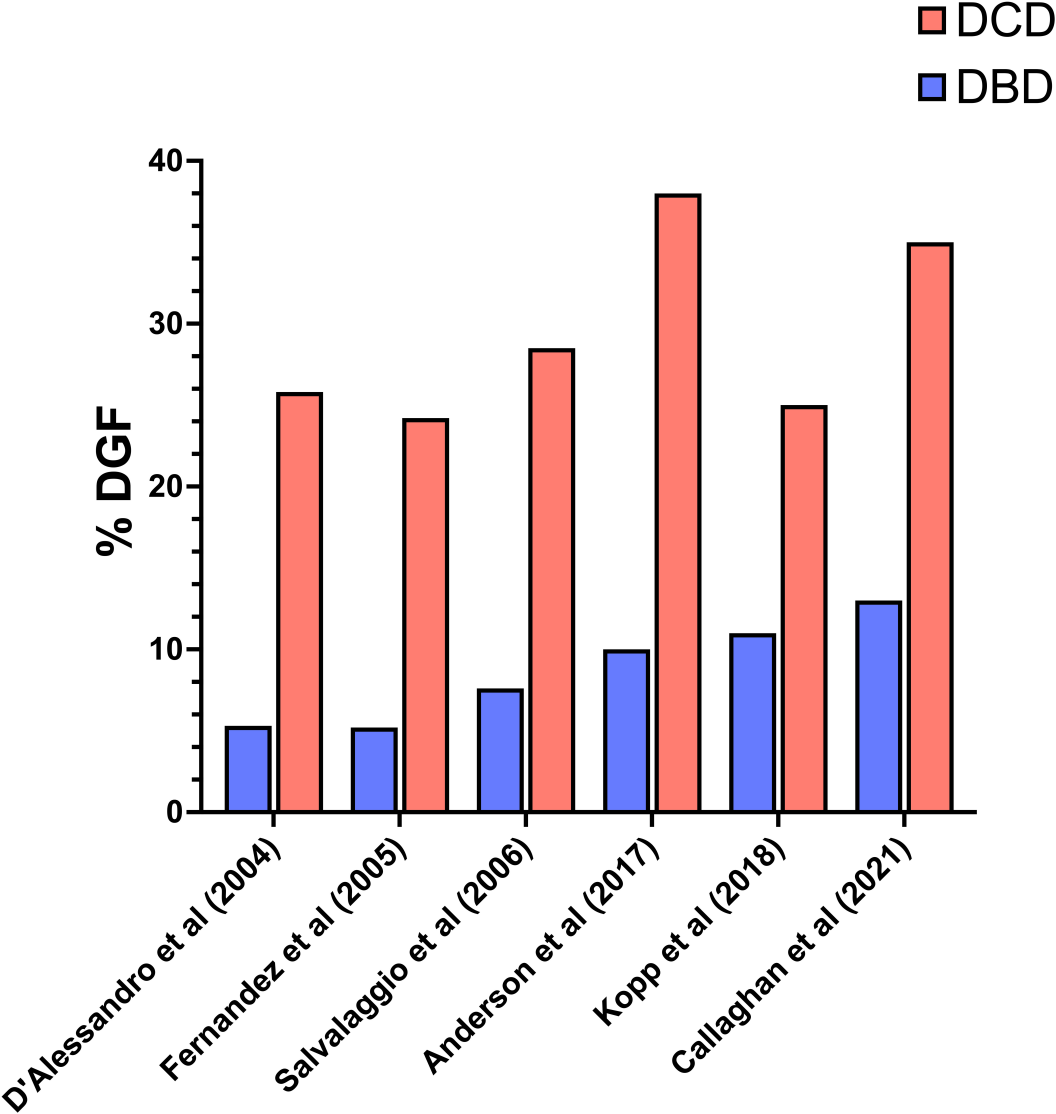
DGF (delayed graft function). Incidence rates of DGF in DCD and DBD pancreatic allografts.

Delayed graft function (DGF) is a significant and concerning complication in simultaneous SPK transplants. The results of eight studies (6, 16, 18, 22, 24–27), were analyzed to compare DGF between DCD and DBD transplants (Figure 4). D'Alessandro et al. (24) conducted a comprehensive analysis comparing outcomes from 31 DCD SPK vs. 455 DBD SPK transplants. DCD pancreas exhibited a higher incidence of DGF (25.8% vs. 5.3%, *p* = 0.01). Similarly, Fernandez et al. (25) found that the incidence of post-transplant DGF was significantly higher in the DCD group (24.3%) compared to the DBD group (5.2%, *P* = 0.0002). Nevertheless, these initial differences resolved within one-month post-transplantation and did not appear to affect long-term graft survival. Other studies such as Salvalaggio et al. (22) in 2006 and Anderson et al. (26) in 2017 report DGF rates of 28.5% and 38% in DCD grafts, respectively, vs. 7.6% and 10% in DBD grafts. Similar trends in more recent investigations by Kopp et al. (6) in 2018, and Callaghan et al. (16) in 2021, showed higher DGF rates in DCD grafts (respectively 25% and 35%) compared to DBD grafts (respectively 11% and 13%). However, contrasting findings by Bleszynski et al. (18) suggest no significant difference in DGF between DCD and DBD transplants, highlighting variability in outcomes. In this study, involving a propensity-matched analysis of 32 DCD and 96 DBD pancreas transplants performed between 2011 and 2020, no instances of delayed graft function (DGF) or primary non-function were observed in either group.

### Other complications

eGFR levels were significantly lower in the DCD group compared to the DBD group at both day 7 and day 14 post-transplant, reflecting potential early renal dysfunction in recipients of DCD grafts. Anderson et al. (26) specifically reported reduced eGFR levels in the DCD cohort. Additionally, Bleszynski et al. reported a higher incidence of insulin resistance in recipients from DCD donors than from DBD donors (18.8% vs. 6.2%) indicating the need for enhanced glucose management in this population (18).

Urinary tract infections (UTIs) within the first-year post-transplant were more frequent in DCD recipients, as highlighted by Fernandez et al. (25), who reported UTI rates of 65% and 59.4% in the DCD group compared to 49% and 37.7% in the DBD group, respectively.

Postoperative bleeding was another significant complication observed more frequently in the DCD group. In 2018, Kopp et al. (6) reported a 38% incidence of postoperative bleeding in DCD pancreas transplant recipients, compared to 11% in the DBD group, necessitating careful perioperative management and monitoring to prevent and manage hemorrhagic complications effectively.

Postoperative pancreatitis, indicated by elevated serum amylase and lipase levels, was significantly more prevalent in the DCD cohort, as noted by Richards et al. (19). More in details, this study investigated 211 simultaneous pancreas and kidney transplants, including 139 from DBD donors and 72 from DCD donors [59 static DCD (sDCD) and 13 normothermic regional perfusion (NRP)]. Recipients of sDCD grafts exhibited higher post-transplant amylase and lipase levels compared to DBD grafts, indicating more severe graft pancreatitis. However, lipase levels were significantly lower in the NRP cohort compared to sDCD, suggesting that NRP may mitigate graft pancreatitis, warranting further investigation. While abscesses, fistulas, and pancreatitis were reported more frequently in the DCD group (19% vs. 4.5% in the DBD group) (27), these differences did not reach statistical significance.

### Warm ischemia time (WIT)

**Figure 5:**
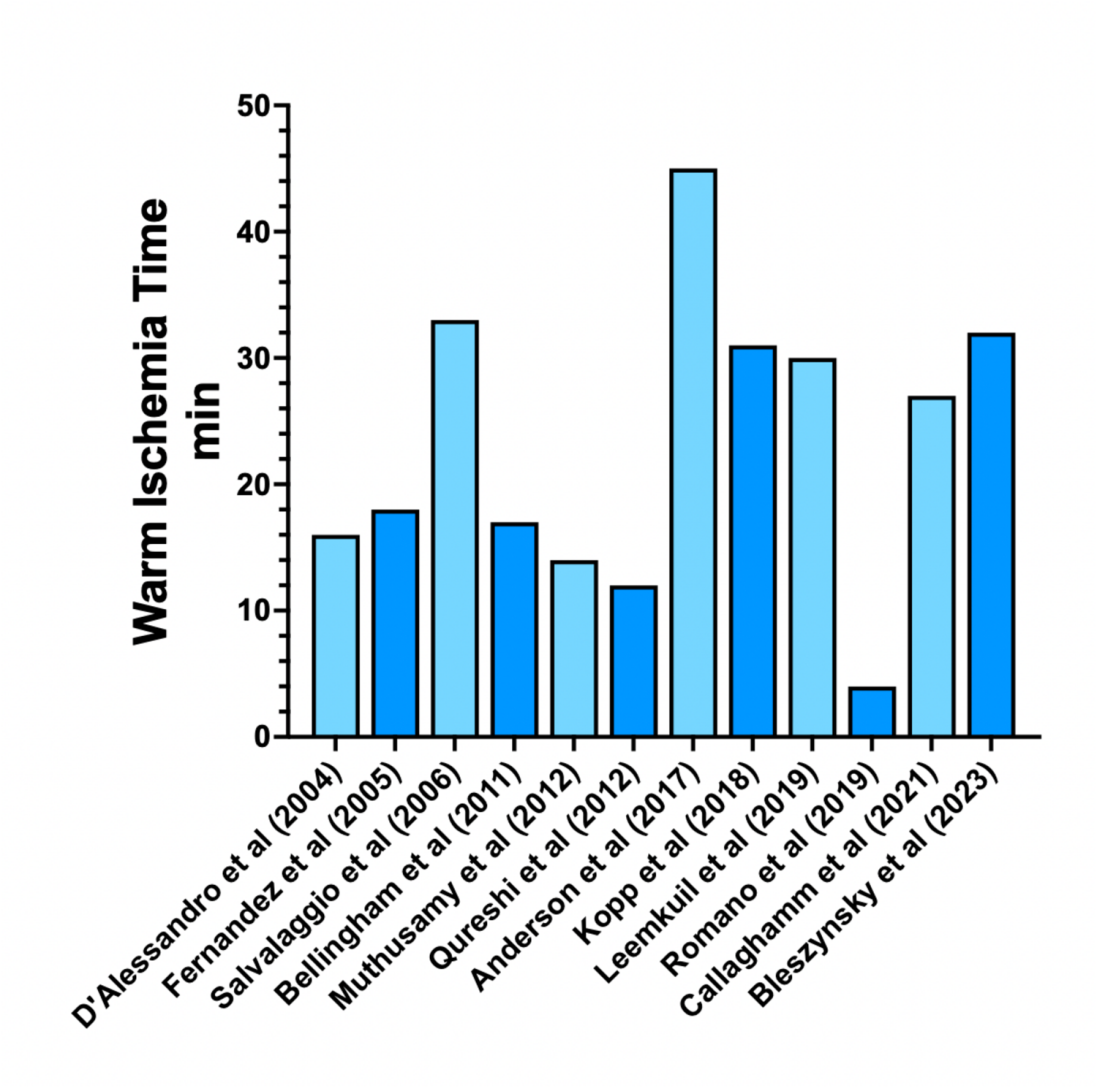
Pancreatic graft warm ischemia time. Mean warm ischemia times for DCD and DBD pancreatic allografts in the literature.

WIT refers to the period extending from the cessation of life-sustaining treatment to the commencement of perfusion with cold preservation fluids. This period can vary significantly (Figure 5), ranging from 4 (27) to a max of 60 min (26). Heterogeneous results were observed when comparing WIT with different endpoints. A mean WIT of 4 min in DCD donors was not associated with a significant difference in the incidence of delayed graft function (DGF) or graft thrombosis in the monocentric study by Romano et al. (27). Most studies reported a median WIT of between 15 and 25 min. Notably, Callaghan et al. (16) showed a median warm ischemic time of 26 min in 403 DCD cases and reported no significant postoperative complications compared to DBD cases. Studies reporting complications related to graft thrombosis have reported variable median warm ischaemia times (WIT). Muthusamy et al. (17) documented a median WIT of 13 min, while Salvalaggio et al. (22) reported a significantly longer median WIT of 30 min. The latter study, representing the cumulative North American experience with DCD donation in SPK transplantation, included over 57 SPK transplants. Despite a higher incidences of DGF, pancreas graft thrombosis and longer hospital stays in the DCD cohort, comparable 5-year allograft survival rates were observed between DCD and DBD donors. The investigators attributed these favorable outcomes to a potentially lower WIT achieved in US centres, facilitated by differences in organ procurement techniques. Specifically, the use of pre-mortem femoral cannulation, administration of heparin and phentolamine, and withdrawal of life support in the operating theatre were identified as key factors contributing to reduced WIT. These findings highlight the critical role of optimized procurement protocols in reducing complications and improving outcomes in DCD pancreas transplantation.

### Glycated haemoglobin (Hb1Ac)

The results obtained from various studies regarding outcomes on post-operative HbA1c levels appear homogenous and show no significant differences between the DCD and DBD groups over varying durations post-transplant. Notably, D'Alessandro et al. (24) reported no significant differences in HbA1c levels at the 6-month mark between the two groups. This finding is consistent with the more recent study by Bleszynski et al. (18), which also reported no significant differences at the 6-month interval. Further studies reinforce this pattern of stability in HbA1c levels. Van Loo et al. (23), Qureshi et al. (28), and Kopp et al. (6) found no significant differences after 1-year of follow-up. Extending the timeline, Anderson et al. (26) and Fernandez et al. (25) reported no significant differences in HbA1c levels up to four years post-transplant, with levels remaining within therapeutic ranges. These findings comfort the idea that DCD transplants seem to achieve equivalent long-term glucose control comparable to those obtained with DBD donors.

### Importance of NRP

NRP involves restoring regional circulation to the abdominal organs at body temperature, thus minimizing ischemic injury and improving organ function before transplantation. The process formally commences upon the confirmation of death. Aortic and vena cava canulation are performed, either surgically or percutaneously, allowing for oxygenation of the blood through an external machine. The oxygenated blood is then perfused to the abdominal organs. An aortic occlusion balloon is placed just over the diaphragm preventing cerebral perfusion. By re-establishing perfusion, NRP reduces the adverse effects of WIT and enhances the quality and viability of DCD pancreases. Its use has demonstrated significant improvements in terms of organ utilization, allograft survival and reduced post-operative complications. The findings were particularly evident in the context of liver (35) and kidney transplants (36). The technique has now been adopted on a wide scale in numerous countries, with France being a notable example where it has been made mandatory for DCD donors. At the moment, only two studies report pancreatic outcomes after DCD donation with NRP. Bekki et al. observed no difference in terms of post-transplantation pancreatic outcomes between DCD and DCD with NRP but only 3 pancreases had been procured in the DCD-NRP group (11). Owen et al., reported results of SPK transplantation from DCD donors using NRP (37). They observed a reduced median ischemic time in the DCD-NRP group in comparison to the non NRP group (9.7 h vs. 10.2 h, *p* = 0.013). Results in term of organ function were similar. Altogether, these studies confirm the safety of NRP use for pancreas procurement among DCD donors, and provide reassuring data that NRP does not preferentially benefit the liver at the expense of other organs. Furthermore, the use of NRP in DCD donors allows for proper assessment of the pancreatic tissue quality *in situ* and improves the quality of pancreas procurement by allowing more precise *in situ* dissection, avoiding iatrogenic injuries to the pancreas. It also reduces reperfusion related bleeding in the recipient. To conclude, the use of NRP is increasing worldwide for DCD donors and will likely contribute to the expansion of the donor pool.

### Machine perfusion for the pancreas, where are we?

Machine perfusion has been successfully implemented for the liver, kidneys, lungs and the heart. However, despite promising experimental results*,* it is not currently used in clinical transplantation. The pancreas is a vulnerable organ with low perfusion and that lacks terminal vascularization, making application of a machine perfusion very challenging. Applying excessive perfusion pressure in the perfusion circuit results in oedema and tissue injuries and conversely, exceedingly low pressure results in inadequate perfusion (38).

Research has been conducted on hypothermic (HMP) and normothermic machine perfusion (NMP). Regarding HMP, several studies demonstrated the feasibility and safety of pancreas preservation until up to 24 h in both human discarded pancreases and large mammal organs (39–41). The results altogether demonstrate that the pancreas maintains preserved endocrine functions after HMP. Prudhomme et al. reported in 2021 the first successful pancreas allotransplantation after 6 h of HMP in a porcine model (42).

NMP allows the pancreas to be perfused at room temperature in optimal conditions while assessment of the organ functions takes place. However, this technique faces critical challenges. Barlow et al. reported the first results using NMP among discarded human pancreases in 2015 (43). Although endocrine secretion of insulin insuline was maintained after normothermic perfusion, extensive histological evidence of necrosis due to organ autodigestion was observed. It is noteworthy to mention that all the studied pancreases had over 13 h of ischemia prior to reperfusion. In 2022, Mazilescu et al. reported their results after pancreas transplantation in a porcine model, using NMP (44). They reported preserved pancreatic endocrine function after transplantation and with only mild necrosis on biopsied specimen and a follow up was 72 h post-transplantation. The adjunction of aproptinin, a dialysis circuit and the application of a low perfusion pressure (25 mmHg) is believed to have contributed to these encouraging results.

Altogether, these very promising results demonstrate the advancements in the field and the desire of the transplant community to move forward with machine perfusion for pancreas allograft preservation. This motivation was confirmed by the European Society for Organ Transplantation (ESOT) who provided in 2023 consensus recommendations on the use of hypothermic machine perfusion for whole pancreas transplantation (45). Despite still facing economic, ethical and organizational challenges, it is very likely that machines perfusion will make their way into clinical transplantation in the following years to come.

### Limitations of the study

The variability in study designs and population demographics, as highlighted in works like Callaghan et al. (16) and Anderson et al. (26), reflects the heterogeneity inherent to pancreas transplantation, a niche surgical field. Differences in outcomes may stem not only from donor type but also from variations in medical practices and patient management strategies across regions and healthcare systems. This makes achieving consistency and clarity in methodological design and data interpretation challenging. Many studies included in this review face limitations, particularly the relatively small sample sizes for DCD transplants compared to DBD (29). This limitation affects the statistical power and may obscure meaningful differences or similarities, as suggested by Leemkuil et al. (20). Furthermore, the lack of standardised post-transplant management protocols introduces variability, with advancements in preservation and perfusion techniques (20, 28), not uniformly accounted for across studies. These factors highlight the need for future research to address these methodological challenges and provide more definitive conclusions about the efficacy and safety of DCD donors in pancreas transplantation.

## Discussion

Pancreas transplantation is the gold standard treatment approach for patients with diabetes mellitus presenting with serious, progressive complications related to their condition and among whom quality of life is deemed unacceptable (1–3). It offers improved metabolic control, eliminating the need for exogenous insulin therapy and may provide additional organ support when combined with kidney transplantation (SKP) in patients with diabetes and ESKD (1). However, the ongoing shortage of suitable donors is a major challenge, particularly for pancreas-only transplantation (PTA), which accounts for less than 10% of all pancreas transplantation worldwide (1, 2). In response, the use of donors after circulatory death (DCD) has emerged as an effective strategy to expand the donor pool. This scoping review highlights the growing potential of pancreas donation after circulatory death (DCD), to address the critical organ shortage.

The equivalency in long-term patient and graft survival rates between DCD and donation after brain death (DBD) donors underscores the viability of DCD as an alternative organ source. This parity could encourage expert transplant centres to expand their acceptance criteria and integrate DCD donors into routine practice, significantly alleviating the shortage of pancreases available for transplantation. Studies conducted between 2011 and 2023 consistently demonstrate that long-term graft survival rates for DCD transplants are comparable to those of DBD transplants with no significant differences in graft survival at 1, 3, 5, and 10 years (16–18, 20). These findings collectively reinforce the growing expertise and confidence within the transplant community in addressing the unique challenges of DCD pancreas transplantation. However, even if DCD pancreas transplantation does not alter long-term graft survival, the increased incidence of early complications in DCD transplants, such as delayed graft function (DGF) and graft thrombosis, underscores the need for optimized clinical protocols and vigilant patient monitoring post-transplant. Multiple studies have demonstrated that these complications manifest at a higher frequency during the early phase of DCD transplants (16, 25). This reflects the preserved long term functional viability of the pancreas irrespective of the donation type. Nevertheless, mitigating these early challenges could requires the integration of advanced preservation techniques such as normothermic regional perfusion (NRP) and hypothermic machine perfusion (HMP) into clinical settings.

These advancements in organ preservation have broader implications for clinical practice and transplantation policies. By improving the viability of DCD grafts, they expand the donor pool and reduce the likelihood of early complications such as graft failure, possibly contributing to long-term transplant success. Importantly, these technologies make it possible to utilize organs that might otherwise have been deemed unsuitable for transplantation (30). The challenges posed by DCD transplants, including the variability of WIT and the potential for increased complications, highlight the importance of optimizing procurement protocols. Several studies (17, 22) demonstrate that shorter WIT, achieved through techniques such as pre-mortem femoral cannulation, administration of heparin, and withdrawal of life support, can significantly reduce the incidence of complications. These findings underscore the critical role of coordinated procurement strategies in improving outcomes for DCD transplants. The successful integration of DCD organs into transplantation practice could also have significant implications for both clinical protocols and organ allocation policies. Encouraging the acceptance and utilization of DCD donors, while maintaining ethical transparency and patient safety, can substantially increase the number of successful pancreas transplantations performed annually. Studies such as those by D'Alessandro et al. and Elmer et al. argue that advancements in preservation technologies and perioperative care are essential for optimizing DCD transplantation outcomes (24, 31). Furthermore, these advancements emphasize the need for specialized training for transplant teams to handle the unique challenges of DCD donation.

Finally, our review identifies several areas for improvement in research and clinical practice. The heterogeneity in study designs, small sample sizes of DCD transplants, and lack of standardized post-transplant management protocols limit the comparability and generalizability of findings. Addressing these limitations through multicentric prospective studies with harmonized methodologies is essential to establish more definitive conclusions about the efficacy and safety of DCD pancreas transplantation (20, 29). The findings of these future studies will have significant implications for organ allocation policies and ethical considerations in organ donation. As the transplantation community seeks to maximize the equitable and efficient use of available organs, it is essential to understand the unique parameters and differences between DCD and DBD donors to drive consistent progress.

Moreover, it should not be forgotten that artificial intelligence (AI) is increasingly transforming organ transplantation (46–49) but remains underutilized in pancreas transplantation, except beyond rejection diagnosis (50). Expanding AI applications to stratify and select DCD donors could optimize donor evaluation, increase the donor pool, and improve outcomes. Tools like predictive modelling and organ quality assessment (OrQA) could address key challenges, paving the way for personalized strategies and enhancing the viability of pancreas transplantation from DCD donors. Encouraging the broader acceptance and utilization of DCD donors, while ensuring ethical transparency and patient safety, has the potential to increase the number of successful pancreas transplants performed annually.

Studies conducted between 2011 and 2023 further support these conclusions, consistently demonstrating no significant disparities in long-term graft survival rates between DCD and DBD transplants. This evidence reinforces the growing confidence in DCD transplantation as a reliable and sustainable approach to addressing organ shortages. In conclusion, while DCD pancreas transplantation presents unique challenges, advancements in preservation techniques and clinical management have significantly improved outcomes, making it a viable option for expanding the donor pool. Continued innovation and rigorous research are necessary to optimize these outcomes further and ensure the successful integration of DCD organs into routine transplant programs. By addressing the current limitations and leveraging advancements in preservation technologies, the transplant community can move closer to alleviating the critical pancreas shortage.

## Data Availability

The original contributions presented in the study are included in the article/Supplementary Material, further inquiries can be directed to the corresponding author/s.
